# Experimental signatures of nodeless multiband superconductivity in a $\hbox {2H-Pd}_{0.08} \hbox {TaSe}_2$ single crystal

**DOI:** 10.1038/s41598-021-92709-8

**Published:** 2021-06-28

**Authors:** Chanhee Kim, Dilip Bhoi, Yeahan Sur, Byung-Gu Jeon, Dirk Wulferding, Byeong Hun Min, Jeehoon Kim, Kee Hoon Kim

**Affiliations:** 1 Department of Physics and Astronomy, Center for Novel States of Complex Materials Research, Seoul National University, Seoul, 08826 Republic of Korea; 2 Center for Artificial Low Dimensional Electronic Systems, Institute for Basic Science, Pohang, 37673 South Korea; 3 Department of Physics, Pohang University of Science and Technology, Pohang, 37673 South Korea; 4 Department of Physics and Astronomy, Institute of Applied Physics, Seoul National University, Seoul, 08826 Republic of Korea; 5 Present Address: The Institute for Solid State Physics (ISSP), The Institute for solid state Physics, The University of Tokyo, Kashiwa, Chiba 277-8581 Japan

**Keywords:** Condensed-matter physics, Condensed-matter physics

## Abstract

In order to understand the superconducting gap nature of a $\hbox {2H-Pd}_{0.08} \hbox {TaSe}_2$ single crystal with $T_{c} = 3.13 \text { K}$, in-plane thermal conductivity $\kappa$, in-plane London penetration depth $\lambda _{\text {L}}$, and the upper critical fields $H_{c2}$ have been investigated. At zero magnetic field, it is found that no residual linear term $\kappa _{0}/T$ exists and $\lambda _{\text {L}}$ follows a power-law $T^n$ (*T*: temperature) with *n* = 2.66 at $T \le \frac{1}{3}T_c$, supporting nodeless superconductivity. Moreover, the magnetic-field dependence of $\kappa _{0}$/*T* clearly shows a shoulder-like feature at a low field region. The temperature dependent $H_{c2}$ curves for both in-plane and out-of-plane field directions exhibit clear upward curvatures near $T_c$, consistent with the shape predicted by the two-band theory and the anisotropy ratio between the $H_{c2}$(*T*) curves exhibits strong temperature-dependence. All these results coherently suggest that $\hbox {2H-Pd}_{0.08} \hbox {TaSe}_2$ is a nodeless, multiband superconductor.

## Introduction

Multiband superconductivity (MBSC), which features multiple superconducting gaps at various Fermi surfaces, has become one of common properties observed in numerous superconductors. The possibility for the MBSC was first discussed in theoretical studies, in which the single-band BCS theory^1^ has been generalized into the case of multi-band superconductivity^2,3^. The first experimental signature was indeed found in early 1980s when a tunneling spectroscopy study revealed two superconducting gaps in a doped $\hbox {SrTiO}_3$ system^4^. Various other experimental probes such as upper critical fields ($H_{c2}$), heat capacity ($C_p$), and thermal conductivity ($\kappa$) measurements have also verified characteristic signatures of the MBSC in the doped $\hbox {SrTiO}_3$ system^5^. The discovery of $\hbox {MgB}_2$ has brought renewed attention on the physics of MBSC as the material exhibits an unusually high superconducting transition temperature ($T_c \simeq 39 \text { K}$) associated with the two BCS-type superconducting gaps. More recently, experimental signatures for the MBSC have been also observed in iron-based superconductors^6^, in which sign-changing, nodeless gaps exhibit as many as five different electron and hole pockets^7^. Moreover, it has been recently suggested that even sulfur hydrides exhibiting $T_c \simeq 203 \text { K}$ at a high pressure $155\text { GPa}$ could be also associated with the MBSC^8,9^. Therefore, investigations on the possible MBSC in various superconducting materials may provide deeper insight for understanding the pairing mechanism and the pairing symmetry, and even a clue to reach a higher $T_c$.

Transition metal dichalcogenides (TMDs) with the chemical formula $MX_2$, where *M* is a transition metal atom (such as Mo, Ta, or Nb) and *X* is a chalcogen atom (such as Se or S), and have been known since 1960s^10^. Atomically thin layers of TMDs, being mostly direct band-gap insulators, can find applications in novel electronic, optical, and spintronic devices due to their high electron mobility^11^. Physical properties of TMDs can be tuned by various physical parameters to exhibit the interplay and the correlation between various electronic orders^12,13^. As a number of layers increase, the direct band gap quenches and metallic behavior emerges. The most common structural form of the three dimensional TMDs resulted from stacking of thin two dimensional layers has either a octahedral (1T) (such as $\hbox {MoS}_2$ or $\hbox {WS}_2$) or a trigonal prismatic (2H) (such as $\hbox {NbS}_2$, $\hbox {NbSe}_2$, $\hbox {TaS}_2$ and $\hbox {TaSe}_2$) coordination of metal atoms. Besides being metallic, they also become superconductors at low temperatures, and often stabilize a CDW state as a competing electronic order^13^.

However, the characteristics of superconductivity observed in various TMDs has not been understood well. As the superconductivity often arises within the CDW ground state, where complex Fermi surfaces composed of multiple 4*d* or 5*d* bands and ligand *p* bands are involved^14^, there is a good possibility of observing MBSC. On the other hand, the study on the MBSC has been limitedly performed in e.g., Nb-based TMDs with relatively high-$T_{c}$ above 6 K; for example, both $\hbox {2H-NbS}_2$ ($T_{c} \simeq 6.1 \text { K}$) and $\hbox {2H-NbSe}_2$ ($T_{c} \simeq 7.2 \text { K}$) were found to host nodeless multiband superconductivity based on various experimental probes such as $C_p$^15,16^, $\kappa$^17^, London penetration depth ($\lambda _\text {L}$)^18,19^, and angle-resolved photoemission spectroscopy (ARPES)^14^. Not only the Nb-based TMDs but also Ti-based TMDs are reported to exhibit two superconducting gaps; in an underdoped $\hbox {1T-Cu}_x \hbox {TiSe}_2$ crystals, two superconducting gaps were necessary to explain the $\lambda _\text {L}$ data from $\mu$SR measurements^20^. On the other hand, $\kappa$ measurements on $\hbox {1T-Cu}_{0.06} \hbox {TiSe}_2$^21^ and $C_p$ measurements of $\hbox {1T-Cu}_x \hbox {TiSe}_2$ single crystals^22^ show only evidences of single-band superconductivity. Therefore, to fully elucidate the true nature of superconductivity, systematic experimental studies are required in each material system.

Except for the aforementioned Nb- and Ti-based TMDs, it is hard to find systematic studies on the nature of superconducting gaps in other TMDs. The Ta-based TMDs including $\hbox {2H-TaSe}_2$ ($T_c \simeq 0.15 \text { K}$) and $\hbox {2H-TaS}_2$ ($T_c \simeq 0.8 \text { K}$) have relatively low $T_c$, which limits experimental feasibility to investigate superconducting gaps. On the other hand, it has been found that intercalation of Pd into $\hbox {2H-TaSe}_{{2}}$ ($T_{c} \simeq 0.15 \text { K}$) increases $T_{c}$ upto as high as 3.3 K with Pd intercalation ratio *x* = 0.08–0.09^23^. Therefore, the enhanced $T_c$ in $\hbox {2H-Pd}_{0.08} \hbox {TaSe}_2$ offers an opportunity to investigate superconducting gap properties and compare them with those of other superconducting TMDs. Furthermore, a recent ARPES study of $\hbox {2H-Pd}_{0.08} \hbox {TaSe}_2$ has revealed that its underlying Fermi surface (FS) at the normal state before the CDW formation undergoes a Lifshitz transition near this particular composition, forming a van-hove singularity. As a result, topology of the underlying Fermi surface at this optimal doping regime is clearly different from that of $\hbox {TaSe}_2$ and becomes qualiatively similar to that of $\hbox {NbSe}_2$^24^. In view of the fact that $\hbox {2H-NbSe}_2$ has exhibited nodeless muliband superconductivity, it is thus worthwhile to investigate the gap nature of $\hbox {2H-Pd}_{{x}} \hbox {TaSe}_2$ with optimal $T_{c}$.

Here, we report systematic studies on the superconducting gap nature in a $\hbox {2H-Pd}_{0.08} \hbox {TaSe}_2$ single crystal with a nearly optimal $T_{c} = 3.13 \text { K}$, based on the measurements of upper critical fields ($H_{c2}$), in-plane London penetration depth ($\lambda _{\text {L}}$), and thermal conductivity ($\kappa$). All these experimental probes coherently suggest that $\hbox {2H-Pd}_{0.08} \hbox {TaSe}_2$ is a nodeless, multiband superconductor.

**Figure 1:**
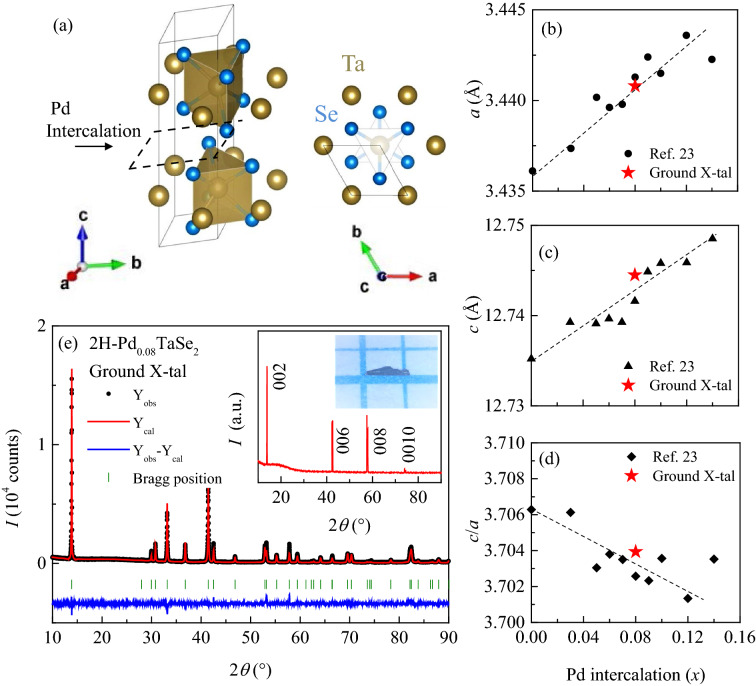
(**a**) The 2H-structure of $\hbox {TaSe}_2$ shows that a pair of two $\hbox {1H-TaSe}_2$ layers form one unit cell. (**b**) and (**c**) show the evolution of lattice parameters *a* and *c*, respectively, with Pd intercalation ratio (*x*), which are replotted from the results by Bhoi et al.^23^. The corresponding values obtained from the refinement of the XRD data on the ground $\hbox {Pd}_{0.08} \hbox {TaSe}_2$ crystals are also plotted as red stars. (**d**) The calculated *c*/*a* ratio based on the data in (**b**) and (**c**). The black dashed lines in (**b**)–(**c**) refer to the linear guide to eyes. The inset of (**e**) shows an XRD pattern measured on the *ab*-plane of a $\hbox {2H-Pd}_{0.08} \hbox {TaSe}_2$ single crystal and a photo of the crystal piece ($\sim$ 1.0 $\times$ 0.2 $\hbox {mm}^2$) lying on a graph paper with one unit of 1 mm. (**e**) An XRD pattern of the ground crystal (black dot), the Rietveld refinement result (red line) with $R_{\text {wp}}$=22.1 and $\chi ^2$=4.06, and their subtracted pattern (blue line) along with the expected XRD peak positions (green ticks).

**Figure 2:**
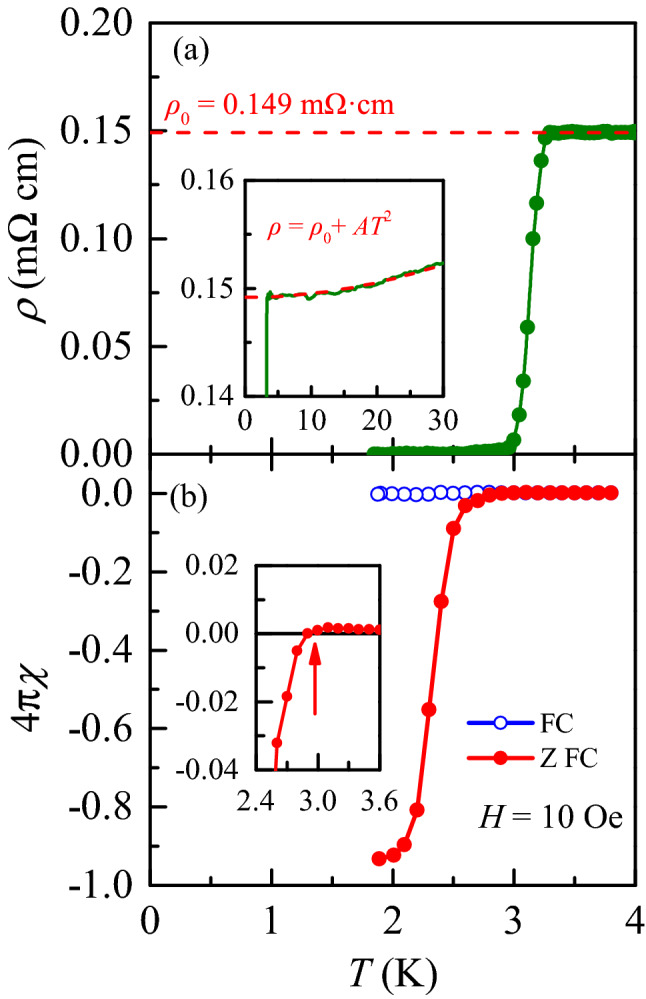
(**a**) Temperature dependence of in-plane resistivity $\rho$ of the $\hbox {2H-Pd}_{0.08} \hbox {TaSe}_2$ single crystal at zero magnetic field. The $\rho$ starts to decrease ($T^{\text {on}}_{c}$) at 3.3 K and goes to zero ($T^{0}_{c}$) at 3 K. The inset shows the resistivity data below 30 K and a power law fitting (red dashed line) with $\rho = \rho _0 + AT^2$, from which $\rho _0$ is estimated as 0.149 $\text {m}\Omega \text {cm}$. (**b**) Magnetic susceptibilities measured at *H* = 10 Oe applied parallel to the *ab*-plane in both zero field cooled (ZFC) and field cooled (FC) conditions. At 1.9 K, $4\pi \chi$ is about 93%, showing nearly full Meissner shielding.

## Results and discussions

### Structure and superconducting properties of a $\hbox {2H-Pd}_{0.08} \hbox {TaSe}_2$ single crystal

Figure 1a presents the 2H-crystal structure of $\hbox {TaSe}_2$, in which a pair of two $\hbox {1H-TaSe}_2$ layers form one unit cell. In each $\hbox {1H-TaSe}_2$ layer, Ta ions are located in the center of a trigonal prism ($D_3^h$ symmetry) created by six Se ions and form a strong in-plane bonding with neighboring Se ions. In the 2H-structure, each $\hbox {1H-TaSe}_2$ layer is rotated by 180$^{\circ }$ along the *c*-axis without in-plane translation, resulting in weak interlayer Se–Se bonding of the van der Waals type along the *c*-axis. Pd ions are intercalated between the Se–Se ions and join a new bonding between neighboring Se ions (see black dashed line). This bonding can contribute to enhance the interlayer interaction even though both *a*- and *c*- lattice constants are known to increase due to the steric nature of Pd intercalation^23^. As a result, according to the lattice parameters replotted from our previous work^23^, both *a* and *c* increase systematically with the Pd concentration *x* (Fig. 1b,c). On the other hand, the *c*/*a* ratio decreases systematically with *x* (Fig. 1d).

For the study in this work,a $\hbox {2H-Pd}_{0.08} \hbox {TaSe}_2$ single crystal has been grown by the chemical vapor transport method, which provides a relatively wide *ab*-plane with a typical lateral area 1.0 mm $\times$ 0.2 mm (see, a photo in the inset of Fig. 1e). When an X-ray beam is shined on the *ab*-plane, only (00*l*) reflections from the X-ray diffraction (XRD) pattern (inset of Fig. 1e) are found, indicating that the crystal layers are well formed along the *c*-axis. To extract accurately the lattice constants of the crystal, many pieces of the $\hbox {2H-Pd}_{0.08} \hbox {TaSe}_2$ single crystals (a total of $\sim$8 mg) collected from the same growth batch were ground and measured by $\theta -2\theta$ scans. An XRD pattern of the ground crystals (black dot) and the Rietveld refinement result (red line) from the FullProf software are shown in Fig. 1e. The refinement considering a preferrential orientation could well reproduce the XRD pattern, resulting in $R_{\text {wp}}$ = 22.1 and $\chi ^2$ = 4.06. The refined *a*- and *c*-values correspond to 3.4408 Å and 12.744 Å, respectively, which are again plotted in Fig. 1b,c together with the calculated *c*/*a* value (Fig. 1d). The lattice constants and the *c*/*a* ratio (red stars) from the single crystal are close to the expected values in the polycrystalline data, indicating successful intercalation of $\sim$8% Pd into the region between the $\hbox {1H-TaSe}_2$ layers.

Figure 2a displays temperature-dependence of in-plane resistivity $\rho$ in the $\hbox {2H-Pd}_{0.08} \hbox {TaSe}_2$ single crystal at zero magnetic field. $\rho$ starts to drop near an onset transition temperature, $T_{c}^{\text {on}} = 3.3 \text { K}$ and goes to zero below $T_{c}^{0} = 3.0 \text { K}$ with a transition width $\delta T_c = T_{c}^{\text {on}}-T_{c}^{0} = 0.3 \text { K}$. $T_c$ is defined by the criterion of 0.5$\rho _N$ ($\rho _N$: normal-state resistivity) to reduce the effects of vortex motion (0.1$\rho _N$ criterion) and superconducting fluctuation (0.9$\rho _N$ criterion)^25^. Note that $\rho$ in a temperature window between 4 and 30 K can be well fitted with a power-law; $\rho$= $\rho _0$ + A$T^2$ with $\rho _0 = 0.149$ m$\Omega \text {cm}$, indicating the Fermi-liquid behavior. Figure 2b shows the temperature dependence of $4\pi \chi$ measured at *H* = 10 Oe ($H \parallel ab$) upon warming after applying zero-field-cooling (ZFC) and field-cooling (FC) conditions. The onset temperature for a diamagnetic signal ($\sim$ 3.0 K) agrees well with $T_{c}^{0}$ = 3.0 K. At 1.9 K, $4\pi \chi$ (*chi*: magnetic susceptibility)reaches − 0.93, exhibiting a nearly complete Meissner shielding expected in a bulk superconductor.

### Evidence for multiband superconductivity from the upper critical fields

#### General behavior of the upper critical fields of a superconductor

A pair breaking under *H* is understood by two major mechanisms, i.e., the Pauli- and the orbital-limiting effect. The former involves difference between the two *chi*’s of superconducting and normal states. A spin paramagnetism lowers the free energy of the normal state relative to that of the superconducting state, thus lowering the critical field for a superconducting transition^26^. This is called as the Pauli-limiting effect. At the Pauli-limited upper critical field at zero temperature ($H_{c2}^{\text {P}}(0)$), a Zeeman splitting energy is same as a superconducting condensation energy, i.e. $\frac{1}{2}\chi _\text {P}(H_{c2}^{\text {P}}(0))^2 = \frac{1}{2} N_F\Delta _0^2$, which yields $H_{c2}^{\text {P}}(0) = \sqrt{N_F/\chi _\text {P}}\Delta _0$. Here, $\Delta _0$ is a superconducting gap at $T = 0$, $\chi _\text {P} = g\mu _{\text {B}}^2N_F$ is the Pauli spin susceptibility in the normal state, $N_F$ is the density of states at the Fermi energy, and *g* is the Lande *g* factor. On the other hand, the orbital-limiting effect is related to supercurrents around the vortices. At the orbital-limited upper critical field at zero temperature ($H_{c2}^{\text {orb}}(0)$), a total kinetic energy of supercurrents around vortex cores exceeds a superconducting condensation energy. This effect is accompanied by an overlap between normal-state vortex cores, leading to $\mu _0H_{c2}^{\text {orb}}(0)=\phi _0/2\pi \xi ^2$, where $\phi _0 = 2.07\times 10^{-7} \text { Oe~cm}^2$ is the flux quantum and $\xi$ is a coherence length^27^.

The Werthamer–Helfand–Hohenberg (WHH) model for a single-band, dirty-limit superconductor involving both of these limiting effects can be applied to determine the $H_{c2}$–*T* relationship^27^;

1 $$\begin{aligned} \ln \frac{1}{t}=\sum \limits _{\nu =-\infty }^{\infty } \left( \frac{1}{\left| 2\nu +1\right| }-\left[ \left| 2\nu +1\right| +\frac{{\bar{h}}}{t}+\frac{\left( \alpha {\bar{h}}/t\right) ^2}{\left| 2\nu +1\right| +\left( {\bar{h}}+\lambda _{\mathrm{so}}\right) /t}\right] ^{-1}\right) , \end{aligned}$$

where $t = T/T_c$, ${\bar{h}}=(4/\pi ^2)(H_{c2}(T)/|dH_{c2}/dT|_{T_c})$, $\alpha = \sqrt{2}H_{c2}^{\text {orb}}(0)/H_{c2}^{\text {P}}(0)$ is the Maki parameter, and $\lambda _{\text {so}}$ is a spin-orbit scattering constant^25,27^. $H_{c2}$ from Eq. (1) for both field directions exhibit linear temperature-dependence just below $T_c$, followed by a saturating behavior with a concave functional form at low temperatures.

However, $H_{c2}$(*T*) curves of multiband superconductors such as $\hbox {MgB}_2$^28^ and several iron-based superconductors^25,29^ display a convex function just below $T_c$. $\hbox {MgB}_2$ even shows a rapid increase of $H_{c2}$ near $T = 0$^28^, which is distinct from the behavior described by Eq. (1). This discrepancy is remedied by the two-band model developed for a dirty-limit superconductor with negligible interband coupling^28^;

2 $$\begin{aligned} a_0\left[ \ln {t}+U\left( \frac{h}{t}\right) \right] \left[ \ln {t}+U\left( \eta \frac{h}{t}\right) \right] +a_2\left[ \ln {t}+U\left( \eta \frac{h}{t}\right) \right] +a_1\left[ \ln {t}+U\left( \frac{h}{t}\right) \right] = 0, \end{aligned}$$

where $a_0= 2(\lambda _{11}\lambda _{22} - \lambda _{12}\lambda _{21})$, $a_1= 1+(\lambda _{11} - \lambda _{22})/\lambda _{0}$ , $a_2= 1 - (\lambda _{11} - \lambda _{22})/\lambda _{0}$, $\lambda _{0} = [(\lambda _{11} - \lambda _{22})^2 + 4\lambda _{12}\lambda _{21}]^{1/2}$, $h = H_{c2}D_1/2\phi _0T$, $t = T/T_c$, and $\eta = D_2/D_1$. $\lambda _{11}$ and $\lambda _{22}$ are intraband BCS coupling constants, $\lambda _{12}$ and $\lambda _{21}$ are interband BCS coupling constants, $D_i$ is in-plane diffusivity of an *i*th band, and $U(x) = \Psi (x+1/2)-\Psi (x)$ where $\Psi (x)$ is the digamma function. This Eq. (2) has successfully described the $H_{c2}$ behavior of numerous multiband superconductors^28,30^.

#### Application to the experimental data

**Figure 3:**
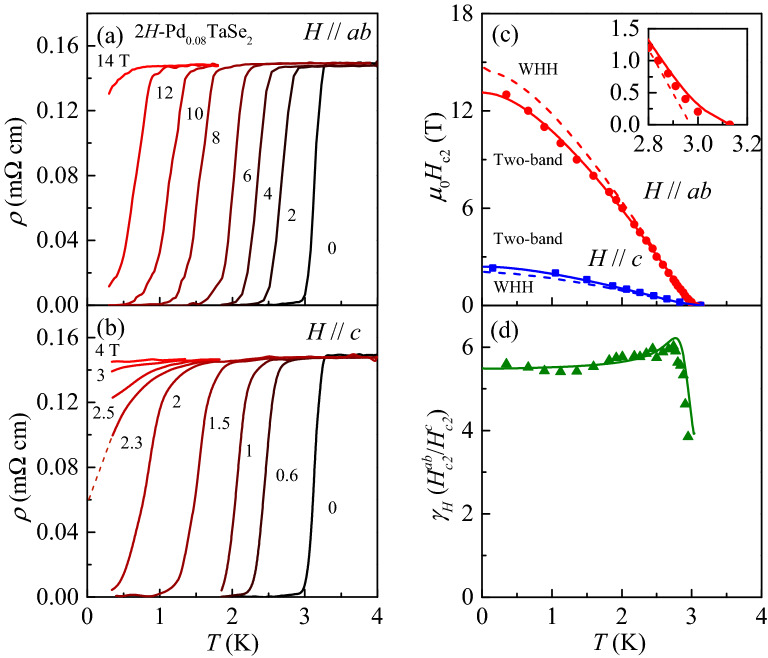
In-plane resistivity $\rho$ of $\hbox {2H-Pd}_{0.08} \hbox {TaSe}_2$ crystal for (**a**) $H \parallel ab$ and (**b**) $H \parallel c$. The dashed line in (**b**) shows a linear extrapolation of $\rho (T)$ to estimate the $T_c$ at $\mu _0H$ = 2.3 T. (**c**) Temperature dependence of upper critical fields for $H \parallel ab$ ($H^{ab}_{c2}$) and $H\parallel c$ ($H^{c}_{c2}$). Also plotted are the best fit results based on the Werthamer–Helfand–Hohenberg (WHH) model (dashed lines) for a single-band superconductor and the two-band model (solid lines). The inset in (**c**) shows an enlarged view of the $H_{c2}^{ab}$ and the fitting lines near $T_c$. (**d**) The anisotropy ratio $\gamma _H = H^{ab}_{c2}/H^{c}_{c2}$ is presented (green solid triangles), exhibiting strong temperature dependence. The solid line is obtained from the fitting curves for the two-band model shown in (**c**).

The $\rho$ curves of $\hbox {2H-Pd}_{0.08} \hbox {TaSe}_2$ are obtained for magnetic fields parallel to the *ab*-plane (*H* $\parallel ab$) and to the *c*-axis (*H* $\parallel$ *c*), as presented in Fig. 3a,b, respectively. In both directions, $\rho$ exhibits negligible magnetoresistance in the normal state so that $\rho _N$ stays nearly at the same value 0.149 m$\Omega \text { cm}$. With increase in *H*, the superconducting transition systematically shifts toward lower temperatures in both directions. Moreover, one can observe the broadening of the superconducting transition with increase of *H*; for example, for $H \parallel c$, $\delta T_c= 0.3\; \text {K}$ at $\mu _0H=0.6$ T increases up to 0.75 K at $\mu _0H = 2$ T. Even for $H \parallel ab$, the increase of transition width is observed; $\delta T_c = 0.3\; \text {K}$ at $\mu _0H = 2$ T increases up to 0.5 K at $\mu _0H = 12$ T. The increasing rate of the transition width is higher for $H \parallel c$ than for $H \parallel ab$. Such anisotroic broadening has been commonly observed in numerous type-II superconductors, indicating that anisotropic thermal fluctuation of the vortex state plays a role in the transition broadening process at a high *H* region.

According to the mean-field theory of type-II superconductors, owing to thermal fluctuation, the vortex-lattice to the normal-state transition at $H_{c2}$ changes into a crossover from the vortex liquid to the normal state, and the vortex-liquid state freezes into the vortex-lattice state at a lower melting field than $H_{c2}$^31^. In a low field region, the extent of the vortex-liquid region is quantatively characterized by the Ginzburg–Levanyuk number, Gi, which is expressed by the material specific paramters as Gi = 0.5 $(8 \pi 2 \lambda ^2_{\text {L}} k_B T_c/\phi ^2 \xi _{c0})^2$, where $k_B$ is the Boltzmann constant, and $\xi _{c0}$ is a coherence length along the *c* axis^31^. At sufficiently high *H* where the cyclotron radius of Cooper pair $r_0 = (\phi _0/2\pi H)^{1/2}$ becomes shorter than the coherence length $\xi _{ab0}$^32^, situation becomes quite different. In the field range $H>$ Gi$H_{c2}^\prime T_c$, the fluctuation broadening is indeed proportional to the field-dependent Ginzburg–Levanyuk number, $\text {Gi}(H) = \text {Gi}^{1/3}[H/(H_{c2}^\prime T_c)]^{2/3}$, where $H_{c2}^\prime = |dH_{c2}/dT|$ is the linear slope of $H_{c2}$ curves near $T_c$^31^. Since $H_{c2}^{ab\prime }=\sim 5.1\text { T/K} > H_{c2}^{c\prime }=\sim 1.1\text { T/K}$ (see, Fig. 3c), the field limit of $\text {Gi} H_{c2}^\prime T_c$ is lower in the case of $H \parallel c$ so that the transition broadening should become larger. Our experimental results are quite consistent with these theoretical consideration, supporting that the transition broadening at high *H* region is mainly caused by the thermal fluctuation of vortex states.

To find a clue on the pair-breaking mechanism, $H_{c2}$ values were determined from the results in Fig. 3a,b. Note that the error bars in the determined $H_{c2}$ values with the 0.5$\rho _N$ criterion are less than the symbol size. In our former study, the $H_{c2}(T)$ curves of a single crystal $\hbox {2H-Pd}_{0.08} \hbox {TaSe}_2$ were investigaed up to 9 T and down to $\sim$ 100 mK^23^. However, for the analysis of *H*-dependent $\kappa$ measurements, precise estimation of $H_{c2}$ extending to higher fields is required so that $H_{c2}^{ab}$ and $H_{c2}^{c}$ of the $\hbox {2H-Pd}_{0.08} \hbox {TaSe}_2$ single crystal are re-investigated here up to 14 T and down to 0.3 K. Based on the $H_{c2}$ curves obtained in Fig. 3c, we first attempted to fit $\mu _0H_{c2}^{ab}(T)$ and $\mu _0H_{c2}^{c}(T)$ with Eq. (1), assuming $\alpha = 0$ (a case for a pure orbital limiting) and $\lambda _{\text {so}} = 0$ (a case without the spin-orbit effect). However, the data could not be fitted well (dashed lines). The $H_{c2}$ curves from Eq. (1) exhibit a linear temperature-dependence just below $T_c$ in both field directions, followed by a saturating behavior with a concave shape at lower temperatures. The fitting results are inconsistent with the $\mu _0H_{c2}^{ab}(T)$ and the $\mu _0H_{c2}^{c}(T)$ curves, both of which display a convex shape just below $T_c$.

We have thus tried to fit the data with the two-band model explained in Eq. (2)^28^. If the interband coupling is too large in a two-band superconductor, the superconducting gap amplitudes of each gap are equalized and it should behave like a single-band superconductor^33^. In our previous heat capacity data on an optimally intercalated $\hbox {2H-Pd}_{0.09} \hbox {TaSe}_2$ polycrystal, we have observed clear evidence for having two distinct superconducting gaps^23^, indicating negligible interband coupling. This suggests that the two-band model in Eq. (2) can be applied in $\hbox {2H-Pd}_{0.09} \hbox {TaSe}_2$. The fitting curves (solid lines) from the two-band model indeed reproduce $\mu _0H_{c2}^{c}(T)$ and $\mu _0H_{c2}^{ab}(T)$ fairly well, supporting the multiband nature of superconductivity even in $\hbox {2H-Pd}_{0.08}$TaSe${_2}$. For $\mu _0H_{c2}^{ab}(T)$, the best fit provides $D_1 = 0.3$ $\hbox {cm}^2$/s, $\eta = 6.5$, $\lambda _{11} = 0.8$, $\lambda _{22} = 0.8$, and $\lambda _{12} = \lambda _{21} = 0.03$ while for $\mu _0H_{c2}^{c}(T)$, the best fit parameters are $D_1 = 1.54$ $\hbox {cm}^2$/s, $\eta = 2.98$, $\lambda _{11} = 0.52$, $\lambda _{22} = 0.52$, and $\lambda _{12} = \lambda _{21} = 0.025$. Eq. (2) provides $\mu _{0} H_{c2}^{c}$(0)=2.45 T and $\mu _{0} H_{c2}^{ab}$(0)=13.1 T. Using the Ginzburg–Landau expression $\mu _{0} H_{c2}^{c}(0)= \phi _0/2\pi \xi _{ab0}^2$ and $\mu _{0} H_{c2}^{ab0}= \phi _0/2\pi \xi _{c0}\xi _{ab0}$, $\xi _{c0}$ and $\xi _{ab0}$ are estimated to be $2.16 \text { nm}$ and $11.6 \text { nm}$, respectively. Note that the $\xi _{c0}$ is greater than the distance between two neighboring $\hbox {TaSe}_2$ layers (*c*/2 = 6.372 Å), indicating that $\hbox {2H-Pd}_{0.08} \hbox {TaSe}_2$ is a three-dimensional anisotropic superconductor.

The multiband effect is also corroborated by temperature dependence of the anisotropy ratio between the upper critical fields, $\gamma _H = H_{c2}^{ab}/H_{c2}^{c}$ as shown in Fig. 3d. Upon temperature being lowered, the $\gamma _H$ values (solid green triangles) increase rapidly near $T_c$ from $\sim$ 4 to reach a maximum value of $\sim$ 6.0 at 2.7 K, and slowly decreases to become a nearly constant value of $\sim$ 5.5 below 1.7 K. This kind of strong temperature dependence in $\gamma _H$ has been similarly observed in other multiband superconductors, e.g. $\hbox {MgB}_2$^28^ and several iron-based superconductors^29,30^, supporting firmly that $\hbox {2H-Pd}_{0.08} \hbox {TaSe}_2$ is also a multiband superconductor.

**Figure 4:**
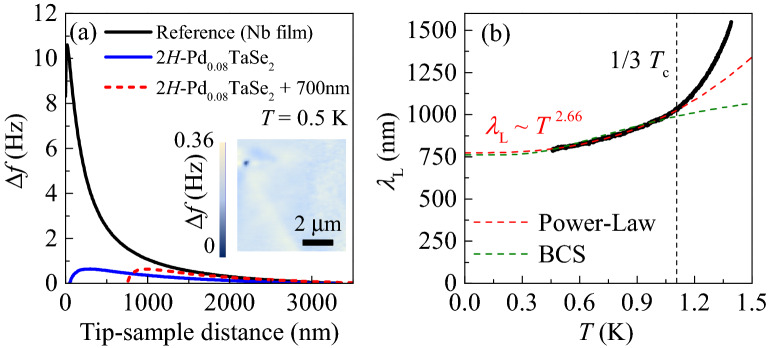
(**a**) Meissner force curves from the $\hbox {2H-Pd}_{0.08} \hbox {TaSe}_2$ single crystal at 0.5 K (blue solid line) and the reference sample (Nb, black solid line) to determine in-plane London penetration depth $\lambda _{\text {L}}$. Using the comparative method (see the text), one can extract the absolute value of $\lambda _{\text {L}}$. The addition of the shifted distance of 700 nm to the black solid line (red dashed line) leads to $\lambda _{\text {L}}(0.5 \;\text {K}) = \lambda _{\text {L,Nb}}(0.5\; \text {K})+z$ = 110 nm + 700 nm = 810 nm. Inset : MFM image obtained at *T* = 0.5 K. (**b**) Temperature dependence of the $\lambda _{\text {L}}$. Below $\frac{1}{3}T_c$, $\lambda _{\text {L}}(T)$ is fitted to both the single-band BCS formula (green dashed line) and the power-law ($AT^n$, red dashed line). The former fails to reproduce the data while the latter fits better the data with the exponent *n* about 2.66, constituting compelling evidence on nodeless, multiband nature of the superconducting gap.

### Evidence for multiband superconductivity: temperature dependence of in-plane London penetration depth

Magnetic force microscopy (MFM) offers a unique opportunity to extract an absolute value of the in-plane London penetration depth ($\lambda _{\text {L}}$)^34^. The so-called comparative method measures a repulsive force between a magnetic tip and a sample and compares it with that between the tip and a standard sample (Nb). The force shifts a resonance frequency of the tip, which is depicted in Fig. 4. The magnetic tip was slowly lowered towards the *ab* surface of the superconducting $\hbox {2H-Pd}_{0.08} \hbox {TaSe}_2$ crystal at $T = 0.5 \text { K}$, which imposes an increasingly strong repulsive Meissner force onto the tip (blue solid line). This Meissner force curve is then compared to that of a well-characterized Nb film with $\lambda _{\text {L,Nb}} = 110 \text { nm}$ (black solid line), measured under the same condition. Any difference in $\lambda _{\text {L}}$ manifests itself as a horizontal shift between the two curves. It is found that once shifted to the higher value by 700 nm, the Meissner force curve well overlaps with that for Nb in a wide range of the tip-sample distance (red dashed line). It is estimated that $\lambda _{\text {L}}$(0.5 K) = $\lambda _{\text {L,Nb}}(0.5 \text { K})+z$ = 110 nm + 700 nm = 810 nm. The inset displays an MFM image of 8 $\upmu$m $\times$ 8 $\upmu$m size, scanned at a tip-sample distance of 300 nm at *T* = 0.5 K. A uniform Meissner force is observed in the entire region except a small defect at the upper left corner, indicating homogeneous superfluid density.

Temperature dependence of $\lambda _{\text {L}}$, $\lambda _{\text {L}} (T)$, is presented at low temperatures below 1.25 K in Fig. 4b. We first attempted to fit $\lambda _{\text {L}}(T)$ with the single-band BCS superconductor model^35^, which is given as $\delta \lambda _{\text {L}}(T) \simeq \lambda _{\text {L}}(0)\sqrt{\pi \Delta _0/2k_BT} \exp \left( -\Delta _0/k_B T\right)$, where $\delta \lambda _{\text {L}}(T) = \lambda _{\text {L}}(T)-\lambda _{\text {L}}(0)$. Following a common practice, the fitting was performed up to 1.04 K ($\simeq \frac{1}{3}T_c$) to minimize the thermal fluctuation effects. The resultant best fit is drawn as a green dashed line, which is clearly inconsistent with the experimental data. Even the obtained parameter $\Delta _0 = 0.60 k_B T_{c}$ is far from the single-band BCS scenario with $\Delta _0 = 1.76 k_B T_{c}$^1^. Hence, the single-band BCS formula cannot explain the $\lambda _{\text {L}}(T)$ behavior.

As the single-band BCS fitting is not satisfactory, we carried out a power-law fitting with $\lambda _{\text {L}}(T) = \lambda _{\text {L}}(0) + AT^n$ (red dashed line), again up to 1.04 K ($\simeq \frac{1}{3}T_c$). The power law fit resulted in a good match with the experimental data when $\lambda _{\text {L}}(0) = 760\text { nm}$ and the exponent $n = 2.66$. We first discuss the meaning of the obtained exponent. The superconductor with a clean *s*-wave gap symmetry in the absence of any nodal structure follows the behavior often producing an exponent $n > \sim \;3--4$ in the power-law fitting scheme^36^. In the presence of impurities, however, a high value of the power exponent often becomes smaller due to the increased quasiparticle density of states inside the superconducting gap^37^. For example, theoretical studies have shown that such modified density of states due to nonmagnetic impurities change the exponential behavior ($n > \sim \;3--4$ ) to $n=2$ in the case of the Fe-based superconductors with sign changing *s*-wave gaps, i.e, $s_{+-}$ state^36,37^ In the experiments of $\hbox {Ba}_{1-x} \hbox {K}_{{x}} \hbox {Fe}_2 \hbox {As}_2$ where a well-defined $s_{+-}$ superconducting gap is likely stabilized, *n*=$\sim$ 2.7–4 has been indeed observed in the range of $0.32 \le x \le 0.47$. Even for a conventional BCS superconductor $\hbox {SrPd}_2 \hbox {Ge}_2$ with $T_c \simeq 2.7 \text { K}$ comparable to our $T_c \simeq 3.1 \text { K}$, the exponent $n = 2.7$, being similar to our results, has been found^38^. Therefore, our exponent $n = 2.66$ support the nodeless superconducting gap structure.

It should be also noted that a clean superconductor with line nodes is theoretically predicted to have $n = 1$, e.g. high-$T_c$ cuprates with the *d*-wave gap symmetry^39^. When nonmagnetic impurity scattering exists in the superconductors with the line node, the exponent *n* was indeed varied from 1.0 toward 2.0 but it was mostly less than 2.0. For example, Zn-doped $\hbox {YBa}_2 \hbox {Cu}_3 \hbox {O}_{6.95}$ showed gradual changes of *n* from 1.13 to 1.75 when the Zn doping into the Cu sites changed from 0 to 0.31%^40^. Therefore, our exponent $n=2.66$ clearly rules out the possibility of superconducting gap state with the nodal lines^36,41^.

To check the validity of the experimentally obtained $\lambda _{\text {L}}(0)$, we herein attempt to calculate $\lambda _{\text {L}}(0)$ using the parameters obtained from the two-band fitting of $H_{c2}$ and the heat capacity measurement^23^. The London equation for a two-band superconductor is given by^42^

3 $$\begin{aligned} \lambda _{\text {L}}^{-2}(0)=\frac{4\pi ^2e^2}{c^2\hbar }\left( N_1D_1\Delta _1+N_2D_2\Delta _2\right) , \end{aligned}$$

where $N_1$ and $N_2$ are the electron densities of states. $\Delta _1$ and $\Delta _2$ are the gap magnitudes. $D_1$ and $D_2$ are the intraband diffusivities. $N_1$, $N_2$, $\Delta _1$ and $\Delta _2$ could be derived from the heat capacity measurements, which yields $N_1$ = 1.51 states/eV f.u., $N_2$ = 0.65 states/cell eV f.u., $\Delta _1$ = 0.49 meV, and $\Delta _2$ = 0.16 meV with a unit cell volume of $V = 87.7 \$\hbox {AA}^3$. From the $H_{c2}$ measurements, the diffusivities are derived as $D_1$ = 1.54 $\hbox {cm}^2$/s, $D_2$ = 4.59 $\hbox {cm}^2$/s, If we combine those parameters, we obtain $\lambda _{\text {L}}(0)\simeq$ 752 nm, which is similar to the measured $\lambda _{\text {L}}(0)= 760 \text { nm}$. This results corroborate that the experimentally determined values such as $\lambda _L(0)$, diffusivities, superconducting gaps, and density of states are consistent each other. Furthermore, the ratio $\lambda _{\text {L}}(0)/\xi _{ab0} = 67 \gg 1$ from the fitting parameter $\lambda _{\text {L}}(0)= 760\text { nm}$ and $\xi _{ab0} = 11.4 \text { nm}$ indicates that type-II superconductivity is realized in $\hbox {2H-Pd}_{0.08} \hbox {TaSe}_2$.

### Evidence for multiband superconductivity: temperature and magnetic-field dependence of thermal conductivity

#### General behavior of thermal conductivity of a superconductor

Thermal conductivity of a material $\kappa$ is described by a sum of each *i*th heat-transferring carrier $\kappa _i$, i.e. $\kappa =\sum _i \kappa _i$. The $\kappa _i$ within a semiclassical approach considering the gapless excitation is generally written as^35^

4 $$\begin{aligned} \kappa _i = \frac{1}{3}c_i v_i l_i, \end{aligned}$$

where $c_i$, $v_i$, and $l_i$ are specific heat, average velocity, and mean free path of the *i*th heat-transferring carrier, respectively. Equation (4) can be applicable to any type of heat carriers such as phonons and electrons.

The phononic thermal conductivity $\kappa _{ph}$ from Eq. (4) is given by

5 $$\begin{aligned} \frac{\kappa _{ph}}{T} = \frac{1}{3T}c_{ph} v_{ph} l_{ph} = \frac{1}{3}\beta v_{ph} l_{ph} T^2, \end{aligned}$$

where $\beta =12\pi ^4 z R/5\theta _D^3$ is related to the coefficient from the phononic specific heat as $c_{ph}=\beta T^3$, $v_{ph}$ is a mean velocity of acoustic phonons, $\theta _D$ is the Debye temperature, *z* is the number of atoms per formula unit (in this case, $z=3$), *R* is the ideal gas constant, and $l_{ph}$ is a phononic mean free path^35^. When phonons are scattered at a rough sample boundary (diffuse scattering limit), it is known that the $l_{ph}$ is limited to the temperature-independent, characteristic sample dimension and thus $\kappa _{ph}/T$ is proportional to $T^2$^43^. However, at low-temperatures, the average phonon wavelength increases to make the surface of given roughness apparently look smoother to result in the so-called specular reflection regime^43^, which renders $l_{ph}$ to be varied with a certain power of *T*, leading to $\kappa _{ph}/T \simeq T^{n-1}$^43,44^.

The electronic thermal conductivity $\kappa _N$ is expressed with the specific heat of electrons $c_{e}=\pi ^2N_Fk_B^2T/3$:

6 $$\begin{aligned} \frac{\kappa _N}{T} = \frac{1}{3T}c_{e} v_F l_e = \frac{1}{9}\pi ^2N_Fk_B^2 v_F^2 \tau = \frac{1}{3}\pi ^2 k_B^2\frac{n\tau }{m^*}, \end{aligned}$$

where $c_{e}$, $v_F$, $l_e$, $\tau$, *n*, and $m^*$ parameters refer to specific heat, Fermi velocity, mean free path, scattering time, carrier density, and effective mass of electrons, respectively. It is noted that $\kappa _N/T$ is independent of *T*. According to the Wiedemann-Franz law^35^, $\rho _N = m^*/ne^2\tau$ leads us to estimate $\kappa _N/T = L_0/\rho _N$ where $L_0=\pi ^2k_B^2/3e^2$ = 2.44 $\times 10^{-8}$ W$\Omega$/$\hbox {K}^{2}$ is the Lorenz number. Then, $\kappa /T$ is as the sum of *T*-independent ($\kappa _N/T$) and $\kappa _{ph}/T \simeq T^{n-1}$ in the normal state.

In a superconducting state, we may describe $\kappa /T$ by a power-law, but the term associated with electrons should be replaced by that for quasiparticles ($\kappa _{0}/T$). The $\kappa _{0}$ of nodeless superconductors is not simply expressed by Eq. (4) as the equation is built on the assumption of gapless states^45^. At $T \ll T_c$ without *H*, the $\kappa _{0}/T$ is given by $\left( \Delta _0/T\right) ^2 \exp \left( -\Delta _0/k_B T\right)$, thereby resulting in $\kappa _{0}/T \rightarrow 0$ as *T* approaches 0 K^35^. This is consistent with the observation that heat is not transferred by the cooper pairs as verified in single-band nodeless superconductors, e.g. Nb^46^ and multiband nodeless superconductors, e.g. $\hbox {2H-NbSe}_2$^17^.

In sharp contrast, nodal superconductors have a non-zero $\kappa _{0}/T$ in the zero temperature limit. This behavior is attributed to the quasiparticles that can be excited at the nodes even at zero temperature^39^. The $\kappa _{0}/T$ is given by $\frac{\pi ^2k_B^2}{3} N(0)v_F^2\tau$ at $T\rightarrow 0$ where *N*(*E*) are the density of states at energy *E*^45^. With the presence of non-magnetic impurities, $\kappa _{0}/T$ is known to approach a finite value, irrelevant to the impurity scattering rate. This is experimentally verified in nodal superconductors, e.g. $\hbox {YBa}_2 \hbox {Cu}_3 \hbox {O}_{6.9}$^45,47^.

The magnetic-field dependence of $\kappa (H)/T$ relies especially on the $\kappa _0(H)/T$ since $\kappa _{ph}/T$ is almost unchanged by *H*. $\kappa _0(H)/T$ can be understood by two mechanisms: the Volovik effect^48^ and the quasiparticle tunneling effect^49^. The former involves a quasiparticle energy shift $\delta E \simeq \mathbf {v_s} \cdot \mathbf {p}$ due to supercurrents around vortices, where $\mathbf {v_s}$ and $\mathbf {p}$ are a velocity of the supercurrents and a momentum of the quasiparticles, respectively. On the other hand, the latter is related to intervortex spacing which is given by $d=\sqrt{\phi _0/H}$^35^. Smaller *d* promotes the tunneling of localized quasiparticles between adjacent vortices^48^; the quasiparticles are then delocalized, leading to a finite $\kappa _0(H)/T$.

In the nodeless single-band superconductors, most of the quasiparticles are confined in a vortex and cannot be subject to the supercurrent outside the vortex. This results in negligible Volovik effect^48^. Therefore, the quasiparticle tunneling effect mainly governs the $\kappa _0(H)/T$ behavior of nodeless single-band superconductors. More specifically, the quasiparticle tunneling effect contributes to increase of $\kappa _0(H)/T$ under magnetic fields. For example, near $H \simeq H_{c2}$, $\kappa _0(H)/T$ is sharply increased due to overlapping of vortices and reaches its normal-state value $\kappa _N/T$. This sharply increasing behavior of $\kappa _0(H)/T$ near $H \simeq H_{c2}$ is often observed in the nodeless single-band superconductors such as Nb^46^ and InBi^50^, and is also applied to the multiband nodeless superconductors^51^.

At low *H* region, on the other hand, nodeless multiband superconductors exhibit a characteristic increase of $\kappa _0(H)/T$, forming a shoulder-like feature^52^. In general, multiband nodeless superconductors can have different gap amplitudes, forming approximately two major gaps $\Delta _{\text {S}}$ and $\Delta _{\text {L}}$, where ‘S’ and ‘L’ denote the smaller and the larger gaps. Under *H* higher than the characteristic field $H^*\simeq \Delta _{\text {S}}^2$, the superconductivity due to $\Delta _{\text {S}}$ is suppressed and the quasiparticles are then delocalized across the $\Delta _{\text {S}}$, resulting in the enhanced $\kappa _0(H)/T$ due to the Volovik effect of the delocalized quasiparticles. Such an enhanced $\kappa _0(H)/T$ at a low *H* region, forming a shoulder-like feature, has been observed in numerous multiband, nodeless superconductors such as $\hbox {MgB}_2$^51^ and several iron-based superconductors^6,53^.

In contrast, the $\kappa _0(H)/T$ of nodal superconductors exhibits behavior distinct from that of nodeless superconductors. The quasiparticles of the nodal superconductors can be stabilized even outside the vortex core because of the gapless quasiparticle excitation at the node. The delocalized quasiparticles then result in shift of their energy and even *N*(*E*) by the Volovik effect. For example, this effect yields $N(E) \rightarrow N(E+\delta E) \simeq (E+\delta E)N_F/\Delta _0$ in *d*-wave superconductors. The energy shift $\delta E$ averaged around the vortex is approximately given by $\simeq \sqrt{H}$^48^, resulting in $\kappa _0(H)/T \simeq N(0) \simeq \sqrt{H}$. This relation was experimentally confirmed by dirty *d*-wave superconductors, e.g. $\hbox {Tl}_{{m}} \hbox {Ba}_{{2}} \hbox {Ca}_{n-1} \hbox {Cu}_{{n}} \hbox {O}_{6+\delta }$ (*m*=2 and *n*=1) (Tl-2201)^54^.

**Figure 5:**
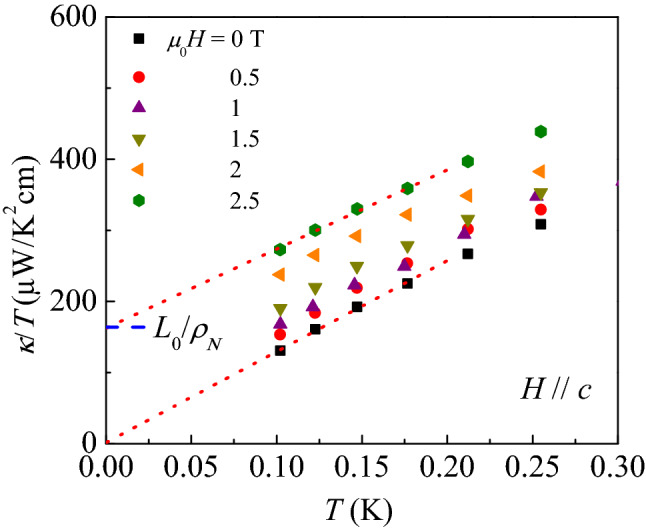
In-plane $\kappa /T$ of the $\hbox {2H-Pd}_{0.08} \hbox {TaSe}_2$ single crystal at various magnetic fields applied along the *c*-axis. The red dotted lines are the fitting curves of $\kappa /T = \kappa _0/T + aT^{n-1}$ with $n = 2$ to the data at $\mu _0H$ = 0 T and 2.5 T below 200 mK. As the magnetic field reaches 2.5 T, which is comparable to $\mu _0H_{c2}^c (0) = 2.45 \text { T}$, $\kappa _0/T$ reaches to the value expected from the Wiedemann–Franz law at the normal state, $\kappa _N/T = L_0/\rho _N$ = 163 $\upmu$W/$\hbox {K}^{2}$ cm (blue dashed line). The magnetic field dependence of extracted $\kappa _0/T$ is summarized in Fig. 6.

**Figure 6:**
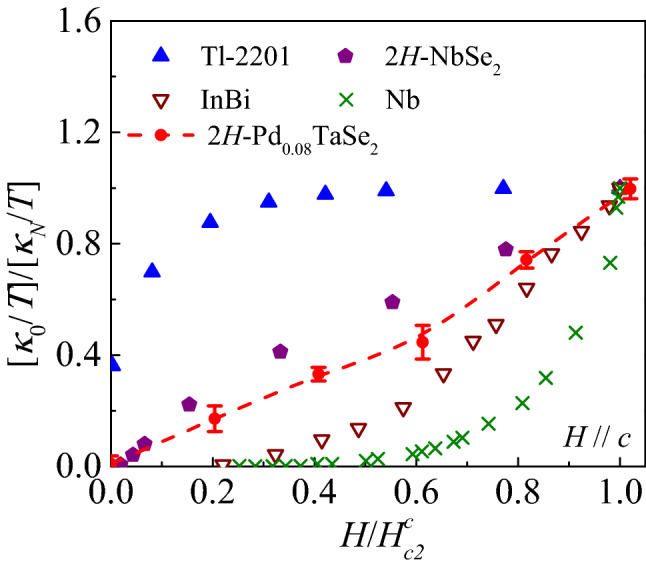
Field dependence of the residual linear term $\kappa _0(H)/T$ of the $\hbox {2H-Pd}_{0.08} \hbox {TaSe}_2$ single crystal. The $\kappa _0(H)/T$ is normalized by the normal-state residual linear term $\kappa _N/T$ = 163 $\upmu$W/$\hbox {K}^{2}$ cm and *H* is normalized by $\mu _0H_{c2}^c(0)$ = 2.45 T. The $\kappa _N/T$ is estimated by the Wiedemann–Franz law and the $\mu _0H_{c2}^c(0)$ is determined from the resistivity measurements (see the text). Error bars correspond to uncertainty in the extrapolation from the power-law fitting. For comparison, $[\kappa _0(H)/T]/[\kappa _N/T]$ of a *d*-wave superconductor $\hbox {Tl}_2 \hbox {Ba}_2 \hbox {CuO}_{6+\delta }$ (Tl-2201)^54^, a multigap nodeless superconductor $\hbox {2H-NbSe}_2$^17^, a dirty nodeless superconductor InBi^50^, and a single nodeless superconductor Nb^46^ are plotted together.

#### Applications to the experimental data

In-plane thermal conductivity $\kappa$ of the $\hbox {2H-Pd}_{0.08} \hbox {TaSe}_2$ single crystal was measured down to 100 mK at various magnetic fields. Figure 5 presents temperature dependence of $\kappa /T$. We fit the data with a generic relation $\kappa /T = \kappa _{0}/T + aT^{n-1}$ to extract $\kappa _{0}/T$ and *n* at zero and finite magnetic fields below 200 mK. At zero magnetic field, $\kappa _{0}$/T = $1.54 \pm 4.68$ $\upmu \text {W}/\text {K}^{2}\;\text {cm}$ and the exponent $n = 2$ are obtained. The deviation of the *n* from 3 immediately supports the occurrence of the specular reflection at the boundary in this low temperature region^43^. More importantly, it should be noted that $[\kappa _{0}/T]/[\kappa _N/T] = 1$% is much smaller than the value expected in a nodal superconductor. Here, $\kappa _{N}/T$ = 163 $\upmu \text {W}/\text {K}^{2}\;\text {cm}$ is the estimated normal state value by the Wiedemann–Franz law and the normal state resistivity $\rho _N = 1.49 \times 10^{-4}\text { }\Omega \; \text {cm}$. Note that the *d*-wave superconductor Tl-2201 has $\kappa _{0}/T = 1.41 \text { mW}/\text {K}^{2}\; \text {cm}$ which is approximately 36% of $\kappa _N/T = 3.95 \text { mW}/\text {K}^{2}\text { cm}$^54^. Therefore, the negligible $\kappa _0/T$ found here thus strongly supports that the superconducting gap of $\hbox {2H-Pd}_{0.08} \hbox {TaSe}_2$ is nodeless.

When a finite magnetic field ($H \parallel c$) is applied, we find that while the power of $n\approx 2$ is almost maintained similar to the zero-field result, the *y*-axis offset corresponding to $\kappa _0/T$ is systematically increased. At $\mu _0H = 2.5 \text { T}$, which is higher than to $\mu _0 H_{c2}^c (0) = 2.45 \text { T}$, we obtain $\kappa _0(2.5\text { T})/T \approx$ $162 \pm 6$ $\upmu \text {W}/\text {K}^{2}\text { cm}$, being comparable to $\kappa _N/T = 163$ $\upmu \text {W}/\text {K}^{2}\text { cm}$. This observation shows that *H* $\approx$ $H_{c2}^c(0)$ restores all the electrons to participate in the heat transfer in accordance with the Wiedemann–Franz law, corroborating that the fully gapped superconducting state is suppressed at the magnetic field close to $\mu _0 H_{c2}^c (0)$^6,55^.

In Fig. 6, we present normalized residual linear terms $[\kappa _0(H)/T]/[\kappa _N/T]$ as a function of normalized magnetic field ($H/H_{c2}^c$). The $[\kappa _0(H)/T]/[\kappa _N/T]$ of a dirty *d*-wave cuprate Tl-2201^54^, a multiband nodeless superconductor $\hbox {2H-NbSe}_2$^17^, a dirty nodeless superconductor InBi^50^, and a clean *s*-wave superconductor Nb^46^ are also plotted for comparison. The fact that $[\kappa _0(0)/T]/[\kappa _N/T]$ is zero for Nb, InBi, and $\hbox {2H-NbSe}_2$ immediately points to the nodeless superconducting state with negligible low-energy quasiparticle excitations. However, the observation of a finite $[\kappa _0(0)/T]/[\kappa _N/T]$ in Tl-2201 evidences the quasiparticle excitation at the nodal superconducting state. Our result for $\hbox {2H-Pd}_{0.08} \hbox {TaSe}_2$ shows nearly zero residual linear term, thereby supporting clearly nodeless superconductivity.

At finite *H*, $[\kappa _0(H)/T]/[\kappa _N/T]$ curves for InBi^50^ and Nb^46^ exhibit sharp increases at $H \simeq H_{c2}$, which is expected in a single-band superconductor. Presence of nodal quasiparticles in the Tl-2201 leads the Volovik effect, which provides significant heat conduction even at zero *H* region^54^. Different from the typical behaviors expected in the single-band *s*- or *d*-wave superconductors, $[\kappa _0(H)/T]/[\kappa _N/T]$ of $\hbox {2H-NbSe}_2$^17^ and $\hbox {2H-Pd}_{0.08} \hbox {TaSe}_2$ exhibit a shoulder-like feature located at  0.2$<H/H_{c2}^c<$ 0.6. This behavior is originated from the multiband superconductivity as discussed in the previous section. Therefore, the shoulder-like feature observed in Fig. 6 supports multiband superconductivity in $\hbox {2H-Pd}_{0.08} \hbox {TaSe}_2$.

It is noted that temperature dependence of $$\kappa /T$$ at various fields has been investigated in another piece of sample (sample2) from the same crystal growth batch. The overall behavior is quite similar to the results in Fig. 5. However, as $$H_{c2}^c$$ curves could not be measured directly on the same piece due to the breaking after the $$\kappa $$ measurements, we have presented the results in the Supplementary Information only. However, once the sample 2 is assumed to have similar $$ H_{c2}^c$$ and $$\kappa _N$$, the sample 2 should still exhibit a shoulder-like feature at a low field region in the $$[\kappa _0(H)/T]/[\kappa _N/T]$$ vs $$H/H_{c2}^c$$ plot. (see, Supplementary Information).

### Determination of cleanness and implications of the multiband superconductivity in the electronic structure

To check whether the sample is in a clean or dirty limit, an intrinsic coherence length $\xi _{0}$ and $l_e$ can be estimated; $\kappa _N/T = \frac{1}{3T}\gamma _0 T v_F l_e =\frac{1}{3}\gamma _0 v_F l_e$ in Eq. (6) leads to the estimation of $l_e = 2.0 \text { nm}$ based on the known values of the normal-state $\kappa _{N}$/T = 163 $\upmu \text {W}/\text {K}^{2}\text { cm}$ from Fig. 5, the Sommerfeld coefficient $\gamma _0$ = 8.56 $\text { mJ}/\text {K}^{2}\text { mol}$ from the specific heat measurement^23^, and $v_F$ = 1.4 $\times$ 10$^5$ m/s from the ARPES^24^. In a BCS superconductor, $\xi _{0}$ is usually close to the BCS coherence length $\hbar v_{F}/\pi \Delta _0$^56^. Here, we estimate $\Delta _0$ as 0.49 meV from the previous specific heat measurement^23^ as $\Delta _0$ is mainly determined by the larger energy gap. Applying these parameters results in $\xi _{0}$ = 60 nm, which is longer than the $\xi _{ab0}$ = 11.6 nm estimated from $H_{c2}$ measurements. In the presence of strong scattering, the electrons can be localized in the scale of $l_e$ and the $\xi _{0}$ can be further reduced by the impurity scattering^35^. The ratio $\xi _{0}/l_e$ thus turns out to be $\sim$ 30, showing that the $\hbox {2H-Pd}_{0.08} \hbox {TaSe}_2$ single crystal is in a dirty limit.

In a recent ARPES study^24^, it is found that $\hbox {2H-Pd}_{0.08} \hbox {TaSe}_2$ at the normal state undergoes a Lifshitz transition with Pd intercalation, resulting in a quite different FS topology as compared with that of $\hbox {TaSe}_2$. In other words, the electron pockets of a dogbone shape, which are originally well separated in $\hbox {TaSe}_2$, have merged to form one connected, bigger Fermi surface in $\hbox {2H-Pd}_{0.08} \hbox {TaSe}_2$ (see, Fig. 3 in Ref.^24^ for details). At the same time, the hole pockets located at the ${\overline{\Gamma }}$ and the ${\overline{\mathrm{K}}}$ points of the crystal momentum in $\hbox {TaSe}_2$ have overall increased their areas. As a result, the Brillouin zone at the normal state is characterized with well-defined hole-pockets and electron-like Fermi surfaces that almost fill up the areas of the whole Brillouin zone. In comparison with the spectra of $\hbox {2H-TaSe}_2$, both electron and hole FSs have increased their areas to result in increased density of states in both electron and hole channels. In this regard, the $T_c$ enhancement with Pd intercalation could be a natural outcome of the increased density of states at the normal states as expected in a BCS superconductor. This further implies that the zone-folding effect caused by the commensurate CDW formation might not affect seriously the overall increase of density of states with Pd intercalation in the underlying electronic structure.

It should be noticed that the resultant FS topology of $\hbox {2H-Pd}_{0.08} \hbox {TaSe}_2$ becomes qualitatively close to that of $\hbox {2H-NbSe}_2$ with $T_{c}\simeq 7.2$ K, which is also known to exhibit nodeless, multiband superconductivity. It is thus inferred that formation of distinctive electron- and hole-Fermi surfaces with large areas at the normal sate should be favorable to the formation of multiband superconductivity in both $\hbox {2H-Pd}_{0.08} \hbox {TaSe}_2$ and $\hbox {2H-NbSe}_2$. A higher $T_{c}\simeq 7.2$ K in 2H-NbSe${_2}$ could be still associated with the difference in the electronic structure. In $\hbox {2H-NbSe}_2$, as compared with the Fermi surface of $\hbox {2H-Pd}_{0.08} \hbox {TaSe}_2$, the density of states seems to be further enhanced with the overall bandwidth decrease due to the 4*d* electrons of Nb. Moreover, a CDW state that can possibly suppress the density of states further is not formed in 2H-NbSe${_2}$.

## Conclusion

In conclusion, we have investigated the superconducting gap structure of a $\hbox {2H-Pd}_{0.08} \hbox {TaSe}_2$ single crystal from upper critical fields, in-plane London penetration depth measurements and thermal conductivity measurements. The upper critical fields in both magnetic field directions show an upward curvature just below $T_c$, and overall shape can be well fitted by the two-band formula in a dirty limit with negligible interband coupling. Moreover, the upper critical field anisotropy exhibits strong temperature dependence. All these behaviors in the upper critical fields constitute strong evidences for the multiband superconductivity.

The London penetration depth, as measured by the magnetic force microscopy with a comparative method, also supports the multiband superconductivity. At $H = 0$, the BCS fitting to $\lambda _{\text {L}}(T)$ results in $\Delta _0 = 0.60 \;k_B T_{c}$, which is smaller than that expected from the BCS theory, $\Delta _0 = 1.76 \;k_B T_{c}$. A power-law fitting to $\lambda _{\text {L}}(T)$ at low temperatures below 1/3 $T_{c}$ provides the exponent $n = 2.66$, which is consistent with the nodeless multiband superconductivity.

Finally, temperature- and field-dependent measurements of thermal conductivity are also consistent with the presence of the nodeless muitiband superconductivity. A vanishingly small residual linear term ($\kappa _0/T$) at zero magnetic field and a shoulder-like feature observed in the plot of $[\kappa _0(H)/T]/[\kappa _N/T]$ verify the scenario of a nodeless multiband superconductivity and rule out the possibility of nodal superconductivity. All these results therefore consistently form compelling evidences that nodeless multiband superconductivity is realized in the single crystal of $\hbox {2H-Pd}_{0.08} \hbox {TaSe}_2$, as similar to the case of $\hbox {2H-NbSe}_2$.

## Methods

Single crystals of $\hbox {2H-Pd}_{0.08} \hbox {TaSe}_2$ were grown by the chemical vapor transport method using $\hbox {SeCl}_4$ as a transport agent as described in our previous report^23^. Room temperature XRD of the crystal was performed by a diffractometer ($\hbox {Empyrean}^{\text {TM}}$, PANalytical). The obtained pattern was refined by the Fullprof software. In-plane resistivity measurements were performed in a Physical Property Measurement System (Quantum Design) by the conventional four probe method. Magnetic susceptibility was measured with a Magnetic Property Measurement System ($\hbox {PPMS}^{\text {TM}}$, Quantum Design). The absolute value of the in-plane London penetration depth was measured in a home-built $^{3}$He-MFM probe, operating inside a 3-axis vector magnet (2–2–9 T in the *x*–*y*–*z* direction)^34^. In-plane thermal conductivity was measured by a standard steady-state two-thermometer, one-heater method in a dilution refrigerator. $\hbox {RuO}_x$ thermometers were carefully calibrated in magnetic fields for the $\kappa$ measurement. For both electrical and thermal transport measurements, contacts were made with silver paste (Dupont 4929$\hbox {N}^{\mathrm{TM}}$). For $\kappa$ measurements, heat current was applied along the *ab*-plane and magnetic field was applied along *c*-axis. We have used the same piece of crystal for XRD, magnetic susceptibility, $\rho$, and $\kappa$ measurements. For $\lambda _{\text {L}}$ measurements, another piece of a crystal from the same batch was used.

## Supplementary information

Supplementary Information.
