# Supplementary material for: Experimental signatures of nodeless multiband superconductivity in a 2H-Pd0.08TaSe2 single crystal
Source: Sci Rep. 2021 Jun 28;11:13383. doi: 10.1038/s41598-021-92709-8 (PMC8239042; doi:10.1038/s41598-021-92709-8)
Supplement: Supplementary file 1 — Supplementary Information. [file 41598_2021_92709_MOESM1_ESM.pdf]

Supplementary information for "Experimental signatures of nodeless multiband superconductivity in a 2H-$Pd_{0.08}TaSe_2$ single crystal"

Chanhee Kim,[1] Dilip Bhoi[a],[1] Yeahan Sur,[1] Byung-Gu Jeon,[1] Dirk Wulferding,[2]

Byeong Hun Min,[1] Jeehoon Kim,[3] and Kee Hoon Kim[1, 4, †]

[1]*Center for Novel States of Complex Materials Research,*

Department of Physics and Astronomy,

Seoul National University, Seoul 08826, Republic of Korea

[2]*Center for Artificial Low Dimensional Electronic Systems,*

Institute for Basic Science, Pohang 37673, Korea

[3]*Department of Physics, Pohang University of*

Science and Technology, Pohang 37673, South Korea

[4]*Institute of Applied Physics, Department of Physics and Astronomy,*

Seoul National University, Seoul 08826, Korea

a Present Address : The Institute for Solid State Physics (ISSP), The Institute for solid state Physics, The University of Tokyo, Kashiwa, Chiba 277-8581, Japan

I. Upper critical field

We have measured temperature dependence of the in-plane resistivity ($\rho(T)$) of 2*H*-$Pd_{0.08}TaSe_2$ for the magnetic field parallel to *c*-axis and along the *ab*-plane. In the main text, due to the clarity, we have only presented $\rho(T)$ from selected magnetic fields. Here, a full dataset of $\rho(T)$ is provided. Upper critical fields determined from the 50% criteria are presented in the main text.

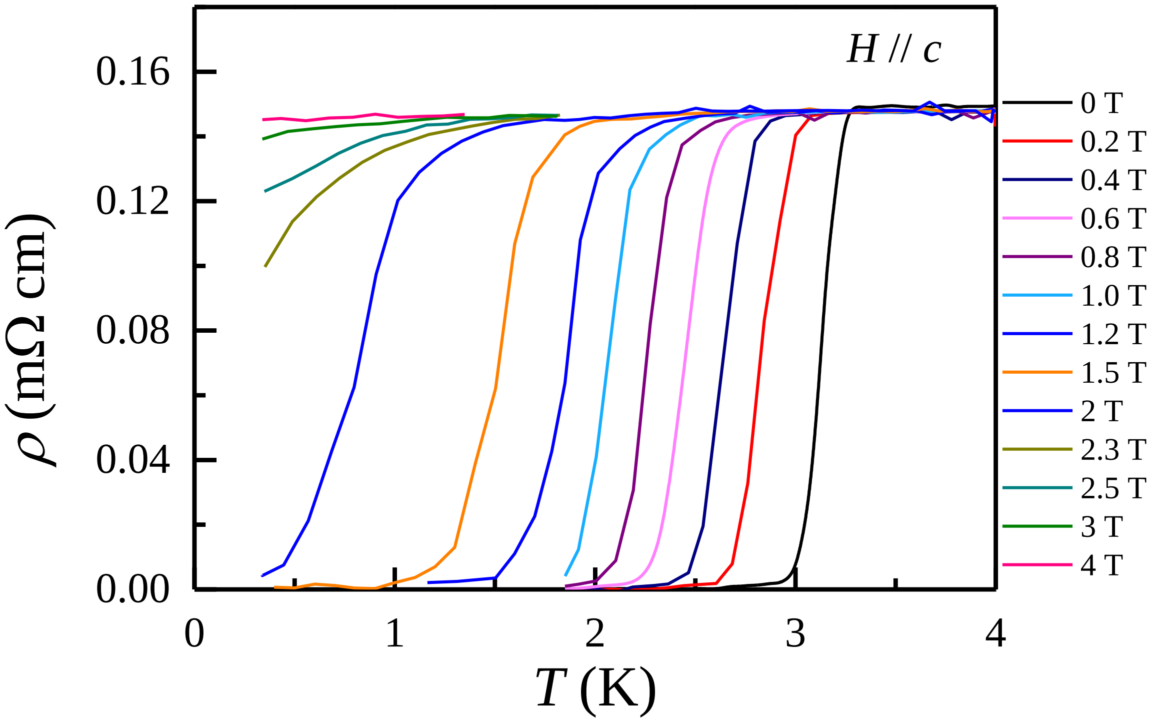

[Figure S1] Temperature dependence of the in-plane resistivity of 2*H*-$Pd_{0.08}TaSe_2$ for the magnetic field applied parallel to *c*-axis.

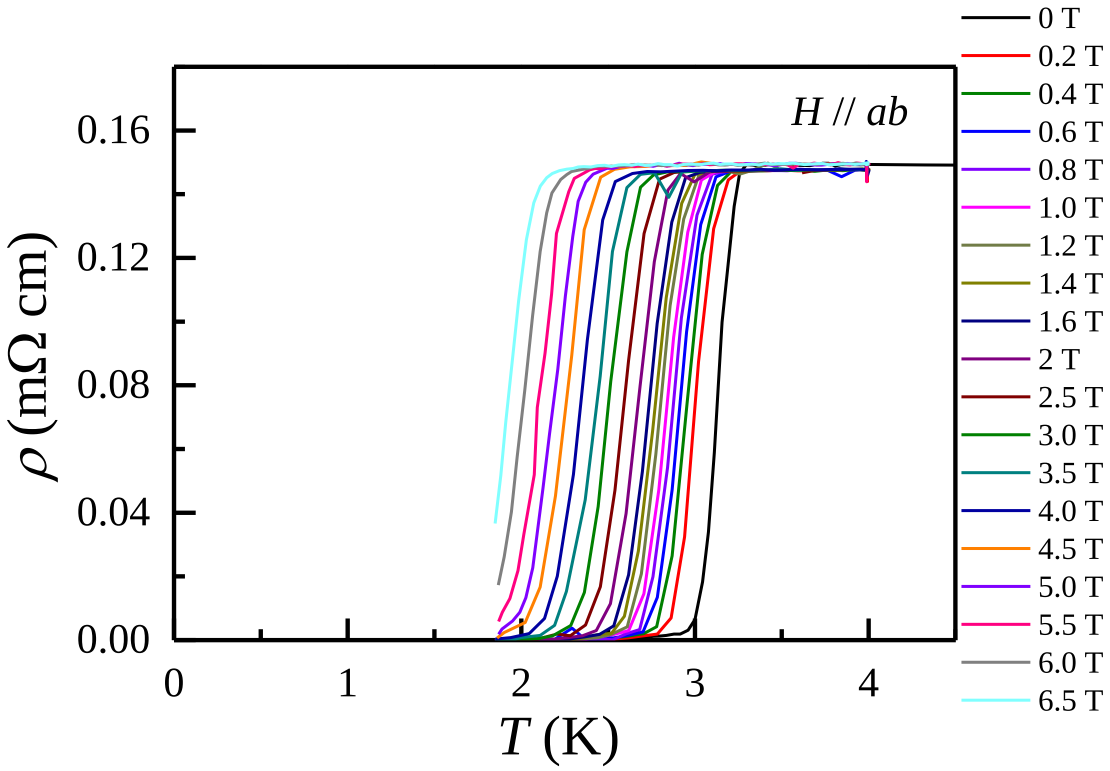

[Figure S2] Temperature dependence of the in-plane resistivity of 2*H*-$Pd_{0.08}TaSe_2$ for the magnetic field up to 6.5 T applied parallel to *ab*-plane.

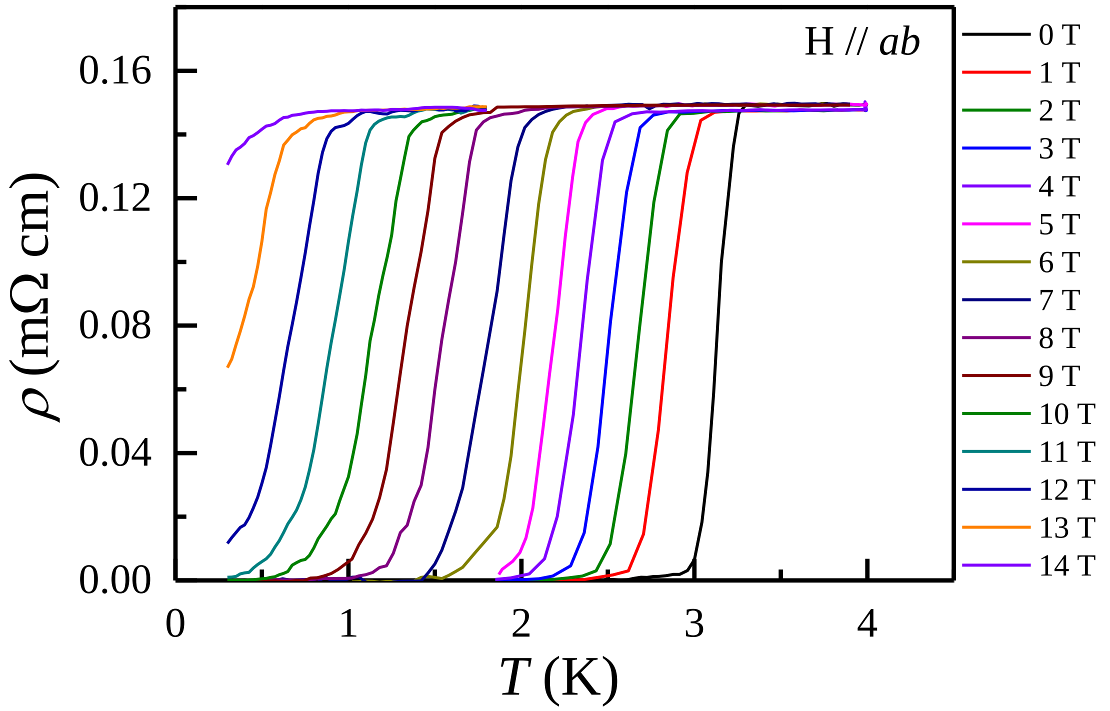

[Figure S3] Temperature dependence of the in-plane resistivity of 2H-$Pd_{0.08}TaSe_2$ for the magnetic field up to 14 T applied parallel to *ab*-plane.

II. Thermal conductivity

To see the reproducibility of our data, the thermal conductivity of another sample piece (sample 2) is measured. The data show a similar behavior as observed in the sample 1.

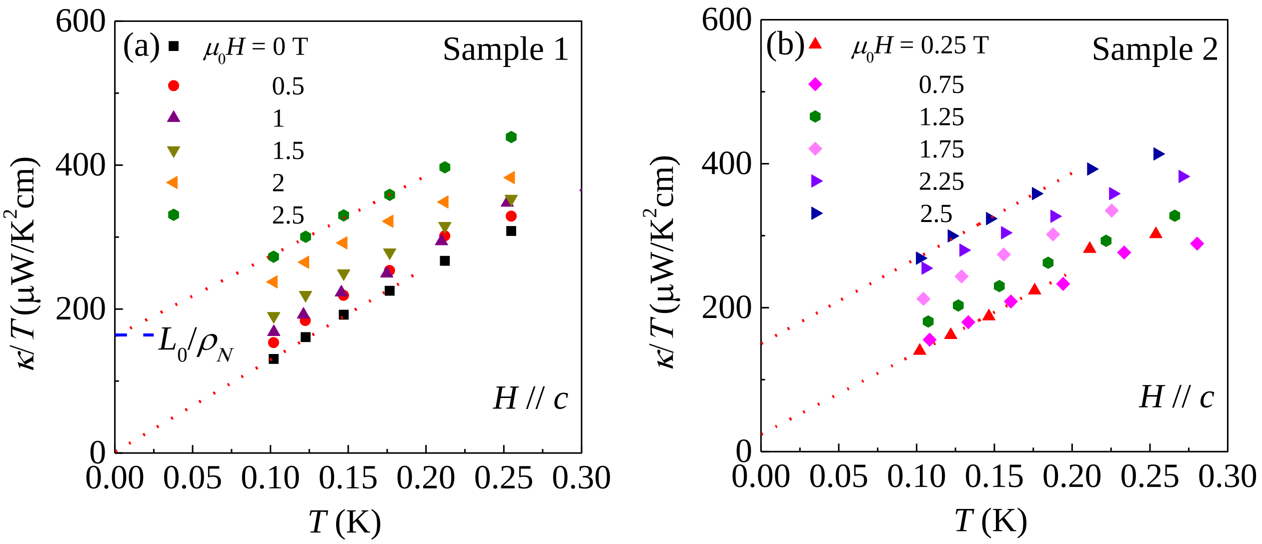

[Figure S4] In-plane κ/T of the 2H-$Pd_{0.08}TaSe_2$ single crystals at various magnetic fields applied along the *c*-axis. The red dotted lines are the fitting curves of $\kappa/T = \kappa_0/T + aT^{n-1}$ with $n = 2$ to the data at $\mu_0 H = 0$ T and 2.5 T for sample 1 and $\mu_0 H = 0.25$ T and 2.5 T below 200 mK for sample 2. For the sample 1, as the magnetic field reaches 2.5 T, which is comparable to $\mu_0 H^c_{c2} \sim 2.45$ T, κ_0/T reaches to the value expected from the Wiedemann-Franz law at the normal state, $\kappa_N/T = L_0/\rho_N = 163$ μW/K^{2}cm (blue dashed line).

Since the H_{c2} of the sample 2 could not be measured, the normalization plot (as in Fig. 5 in the main text) could not be accurately determined. However, at the $\mu_0 H = 2.5$ T, $\kappa_0(2.5\ \mathrm{T})/T$ are found to be 162 ± 6 μW/K^{2}cm and 150 ± 5 μW/K^{2}cm for sample 1 and sample 2, respectively, thereby resulting in similar residual thermal conductivity values in both samples. Therefore, in Fig. S5, we plot $[\kappa_0/T]/[\kappa_N/T]$ of sample 2, assuming that its H_{c2} is 2.45 T and κ_N/T =150 ± 5 μW/K^{2}cm at the zero temperature limit.

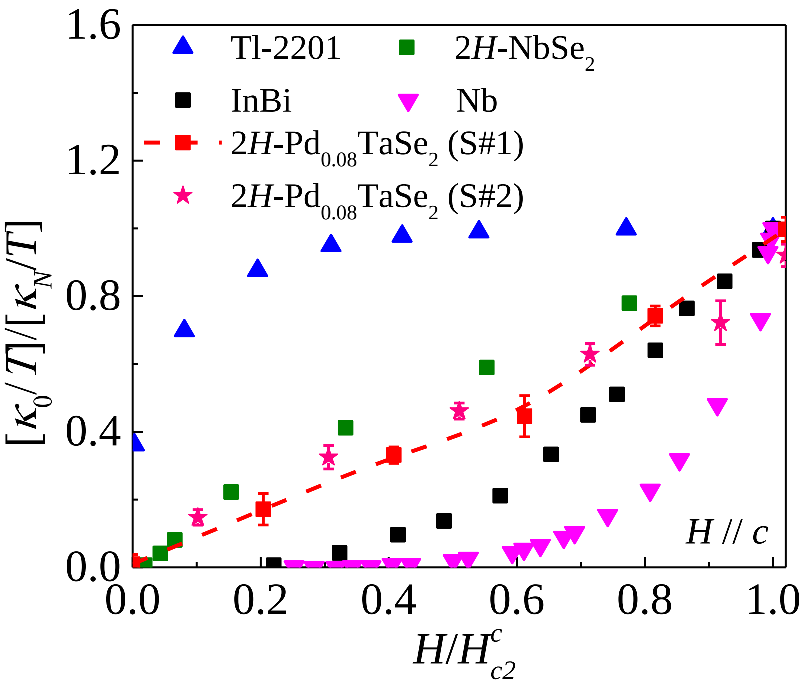

[Figure S5] Field dependence of the residual linear term $\kappa_0(H)/T$ of the 2H-$Pd_{0.08}TaSe_2$ single crystal. In the main panel, for sample 1 (S#1), the $\kappa_0(H)/T$ is normalized by the normal-state residual linear term, $\kappa_N/T = L_0/\rho_N$ = 163 μW/K²cm and $\mu_0 H$ is normalized by $\mu_0 H^c_{c2}$ = 2.45 T. The κ_N/T is estimated by the Wiedemann-Franz law and the $\mu_0 H^c_{c2}(0)$ is determined from the resistivity measurements. For sample 2 (S#2), the data are plotted, assuming that $\mu_0 H^c_{c2}$ is 2.45 T and κ_N/T = 150 μW/K²cm at the zero temperature limit. Error bars correspond to uncertainty in the extrapolation from the power-law fitting. For comparison, $[\kappa_0(H)/T]/[\kappa_N/T]$ of a *d*-wave superconductor $Tl_2Ba_2CuO_{6+\delta}$ (Tl-2201) [1], a multigap nodeless superconductor 2H-$NbSe_2$ [2], a dirty nodeless superconductor InBi [3], and a single nodeless superconductor Nb [4] are plotted together.

In Figure S5, at H/H_{c2} = 0.1, the normalized residual linear term is already 0.17, which is much higher than the value expected in a typical single-band superconductor. Combining the fact that the negligible $[\kappa_0/T]/[\kappa_N/T]$ at zero field for sample 1, we argue that our single crystal is a nodeless, multiband superconductor similar to 2*H*-$NbSe_2$.

III. The relative positions of the individual atoms in the 2H-$Pd_{0.08}TaSe_2$ crystal

Compound	Atom	Relative atomic positions		
		X	Y	Z
2H-$Pd_{0.08}TaSe_2$ (this work)	Ta	0	0	0.25
	Se	0.33	0.66	0.11899
2H-$TaSe_2$ [5]	Ta	0	0	0.25
	Se	0.33	0.66	0.118

[Table S1] The relative atomic positions of the Ta and Se atoms in 2H-$Pd_{0.08}TaSe_2$ single crystal. The atomic positions were obtained by the Rietveld refinement of the XRD data on ground single crystals of 2H-$Pd_{0.08}TaSe_2$ (see, Fig. 1(e)). The relative atomic positions of a pristine 2H-$TaSe_2$ crystal are shown for comparison.

Due to the location of Pd in the layer of van der Waals interaction between the two 1H-$TaSe_2$ layers and small amount (~8 %), the refinement was not sensitive to the variation of Pd ions or location within the layer; namely, it was not possible to locate the exact Pd position inside the intercalated layer from the Rietveld refinement. However, we could obtain the relative positions of Ta and Se atoms in the 2H-$Pd_{0.08}TaSe_2$ crystals, as shown in Table S1. While the relative positions of X and Y did not change due to the crystal symmetry, the Se Z position exhibited a slight increase from 0.118 to 0.11899 compared to the pristine compound. This results in a slight decrease of the Ta-Se distance along the z-direction within the 1H-$TaSe_2$ layer, which is understood as due to the intercalation of Pd ions between the layers.

IV. References

[1] C. Proust, E. Boaknin, R. W. Hill, L. Taillefer, and A. P. Mackenzie, “Heat transport in a strongly overdoped cuprate: Fermi liquid and a pure d-Wave BCS superconductor,” Phys. Rev. Lett. **89**, 147003 (2002)

[2] E. Boaknin, M. A. Tanatar, J. Paglione, D. Hawthorn, F. Ronning, R. W. Hill, M. Sutherland, L. Taillefer, J. Sonier, S. M. Hayden, and J. W. Brill, “Heat conduction in the vortex state of $NbSe_2$: evidence for multiband superconductivity,” Phys. Rev. Lett. **90**, 117003 (2003).

[3] J. O. Willis and D. M. Ginsberg, “Thermal conductivity of superconducting alloy films in a perpendicular magnetic field,” Phys. Rev. B **14**, 1916 (1976).

[4] J. Lowell and J. B. Sousa, “Mixed-state thermal conductivity of type II superconductors,” J. Low Temp. Phys. **3**, 65 (1970).

[5] B. E. Brown, D. J. Beerntsen, “Layer structure polytypism among Niobium and Tantalum selenides,” Acta. Cryst. **18**, 31 (1965)
